# Supplementary material for: Content-rich biological network constructed by mining PubMed abstracts
Source: BMC Bioinformatics. 2004 Oct 8;5:147. doi: 10.1186/1471-2105-5-147 (PMC528731; doi:10.1186/1471-2105-5-147)
Supplement: Additional File 2 — The original results of the above study (non-essential files are deleted to keep the file size under the limit set by BMC bioinformatics). [file 1471-2105-5-147-S2.bz2 › chilibotAdditionalFile2/dip05/181ID1702217E621/html/CSF2RB_CSF2.html]

 


 **CSF2RB** and **CSF2** 
  
Found 2 abstracts in PubMed, retrieved 2.  
 

 What does Google say? 
 PDF only 
| .edu only 

---

**Interactive relationship** (e.g. stimulation, inhibition, etc)

**Non-interactive relationship** (e.g. studied together, co-existance, homology, etc.)

- The gene for the beta chain of the human GM CSF  [ **CSF2** ]  receptor  **CSF2RB**  has been mapped to chromosome 22 by PCR analysis of a series of human x rodent somatic cell hybrids.  Ref: 1424804 Cytogenet Cell Genet, 1992
- Localization of the human GM CSF  [ **CSF2** ]  receptor beta chain gene  **CSF2RB**  to chromosome 22q12.2 >q13.1.  Ref: 1424804 Cytogenet Cell Genet, 1992
